# Integrative multiomics analysis of human atherosclerosis reveals a serum response factor‐driven network associated with intraplaque hemorrhage

**DOI:** 10.1002/ctm2.458

**Published:** 2021-06-27

**Authors:** Han Jin, Pieter Goossens, Peter Juhasz, Wouter Eijgelaar, Marco Manca, Joël M. H. Karel, Evgueni Smirnov, Cornelis J. J. M. Sikkink, Barend M. E. Mees, Olivia Waring, Kim van Kuijk, Gregorio E. Fazzi, Marion J. J. Gijbels, Martina Kutmon, Chris T. A. Evelo, Ulf Hedin, Mat J. A. P. Daemen, Judith C. Sluimer, Ljubica Matic, Erik A. L. Biessen

**Affiliations:** ^1^ Department of Pathology School for Cardiovascular Diseases (CARIM) Maastricht UMC+ Maastricht The Netherlands; ^2^ PJ Consulting Natick Massachusetts USA; ^3^ Department of Data Science and Knowledge Engineering Maastricht University Maastricht The Netherlands; ^4^ Department of Vascular Surgery Zuyderland Medical Centre Heerlen The Netherlands; ^5^ Department of Surgery Maastricht UMC+ Maastricht The Netherlands; ^6^ Department of Medical Biochemistry Experimental Vascular Biology Amsterdam UMC Amsterdam The Netherlands; ^7^ School for Oncology and Developmental Biology (GROW) Maastricht University Maastricht The Netherlands; ^8^ Department of Bioinformatics (BiGCaT) Maastricht Centre for Systems Biology (MaCSBio) Maastricht University Maastricht The Netherlands; ^9^ Department of Molecular Medicine and Surgery Karolinska Institute Solna Sweden; ^10^ Department of Pathology Amsterdam Cardiovascular Sciences Amsterdam UMC Amsterdam The Netherlands; ^11^ BHF Centre for Cardiovascular Science (CVS) University of Edinburgh Edinburgh Scotland; ^12^ Institute for Molecular Cardiovascular Research RWTH Aachen University Aachen Germany

**Keywords:** carotid atherosclerosis, multiomics integration, proteomics, transcriptomics

## Abstract

**Background:**

While single‐omics analyses on human atherosclerotic plaque have been very useful to map stage‐ or disease‐related differences in expression, they only partly capture the array of changes in this tissue and suffer from scale‐intrinsic limitations. In order to better identify processes associated with intraplaque hemorrhage and plaque instability, we therefore combined multiple omics into an integrated model.

**Methods:**

In this study, we compared protein and gene makeup of low‐ versus high‐risk atherosclerotic lesion segments from carotid endarterectomy patients, as judged from the absence or presence of intraplaque hemorrhage, respectively. Transcriptomic, proteomic, and peptidomic data of this plaque cohort were aggregated and analyzed by DIABLO, an integrative multivariate classification and feature selection method.

**Results:**

We identified a protein‐gene associated multiomics model able to segregate stable, nonhemorrhaged from vulnerable, hemorrhaged lesions at high predictive performance (AUC >0.95). The dominant component of this model correlated with αSMA^−^PDGFRα^+^ fibroblast‐like cell content (*p* = 2.4E‐05) and Arg1^+^ macrophage content (*p* = 2.2E‐04) and was driven by serum response factor (SRF), possibly in a megakaryoblastic leukemia‐1/2 (MKL1/2) dependent manner. Gene set overrepresentation analysis on the selected key features of this model pointed to a clear cardiovascular disease signature, with overrepresentation of extracellular matrix synthesis and organization, focal adhesion, and cholesterol metabolism terms, suggestive of the model's relevance for the plaque vulnerability. Finally, we were able to corroborate the predictive power of the selected features in several independent mRNA and proteomic plaque cohorts.

**Conclusions:**

In conclusion, our integrative omics study has identified an intraplaque hemorrhage‐associated cardiovascular signature that provides excellent stratification of low‐ from high‐risk carotid artery plaques in several independent cohorts. Further study revealed suppression of an SRF‐regulated disease network, controlling lesion stability, in vulnerable plaque, which can serve as a scaffold for the design of targeted intervention in plaque destabilization.

## Graphical abstract

1. This multiomics analysis identified a cardiovascular signature in carotid atherosclerotic lesions, which provides excellent stratification of low‐/high‐risk carotid plaques.
2. This study highlights the advantages of multiomics analysis in terms of model robustness, biological significance, and clinical translatability.
3. The prediction model pointed to an SRF‐regulated disease network providing valuable new insights that expedite the design of targeted intervention in plaque rupture.

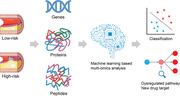

## 1 INTRODUCTION

Atherosclerosis and associated cardiovascular diseases remain one of the leading causes of death worldwide.^1^ In the clinically relevant stage, atherosclerotic lesions may transition from a stable, low‐risk to an unstable phenotype, at high risk of causing acute ischemic events, such as myocardial infarction and ischemic stroke.^2^ Histopathology and experimental studies and more recently, genome‐wide association studies (GWAS) and extensive omics searches have considerably enhanced our understanding of this disease's pathogenesis.^3^

So far, the latter studies have largely relied on global profiling of mRNA expression or protein abundance in plaque tissue and were primarily designed to dissect differential expression, leading amongst others to the identification of plaque‐contained biomarkers correlating with future cardiovascular events (e.g., osteopontin).^4^, ^5^, ^6^ For instance, based on mRNA expression data from the Biobank of Karolinska Endarterectomies (BiKE) cohort,^7^ Diez et al. were able to identify novel gene networks operating in the atherosclerotic plaque.^8^

A limitation of such global approaches is that they often fail to reveal detailed information about a particular cellular component in plaques. Laser‐capture microscopy^9^, ^10^ and single‐cell RNA sequencing‐based analysis of atherosclerotic plaque macrophages^11^, ^12^, ^13^ bypassed this pitfall. These studies allowed assessment and deconvolution, respectively, of the expression profiles of relevant plaque‐contained subsets, and for the latter confirmed the strong proinflammatory nature of non‐foamy plaque macrophages.^13^

However, for indepth dissection of critical processes in plaque, mere transcriptional profiling may fall short incorporating net biosynthesis and protein activity, whereas proteomics profiling alone will bias toward most abundant, rather than most relevant proteins, implying that both scales have serious shortcomings.^14^ Moreover, protein and mRNA profiles are notorious for their rather poor mutual correlation,^15^, ^16^ complicating the translation of genomics findings to clinical practice. Therefore, it is important to interrogate plaques by multiple omics and to evaluate the integrated model for biological and/or clinical implications.^17^ To this end, significant efforts have already been made, and a few recent studies have illustrated the potential of such approaches.^18^, ^19^, ^20^ Applying differential expression and intersect analyses^18^ and/or linking omics with genetics data,^19^, ^20^ these studies largely ignored mutual interaction of gene and/or proteins, and, for the former, did not truly integrate the data scales in a multivariate manner.^21^

Therefore, in the current study we set out to integrate the protein, peptide, and mRNA makeup of carotid artery lesions from the Maastricht Human Plaque Study (MaasHPS)^22^ of symptomatic carotid endarterectomy (CEA) patients. We interrogated a total of 42 carotid artery lesions, based on the presence/absence of intraplaque hemorrhage (IPH), a hallmark of plaque stability and event risk.^23^, ^24^ By integrative machine learning^25^ we were able to identify a composite disease stage classifier, the performance of which was benchmarked against single‐omics derived classifiers using only mRNA expression or protein/peptide abundance and validated in several independent plaque datasets (GEO accession numbers: GSE28829, GSE43292, and GSE21545).

## 2 METHODS

### 2.1 Sample collection and classification

Atherosclerotic plaque samples obtained during carotid endarterectomy from 24 symptomatic patients were collected. The endarterectomy specimens were cut into parallel, transverse segments of 5‐mm thickness. Each alternating segment was snap‐frozen in liquid nitrogen and stored at −80°C, their flanking segments fixed for 24 h in formalin, decalcified for 4 h before processing and embedding in paraffin for histological evaluation.

**FIGURE 1:**
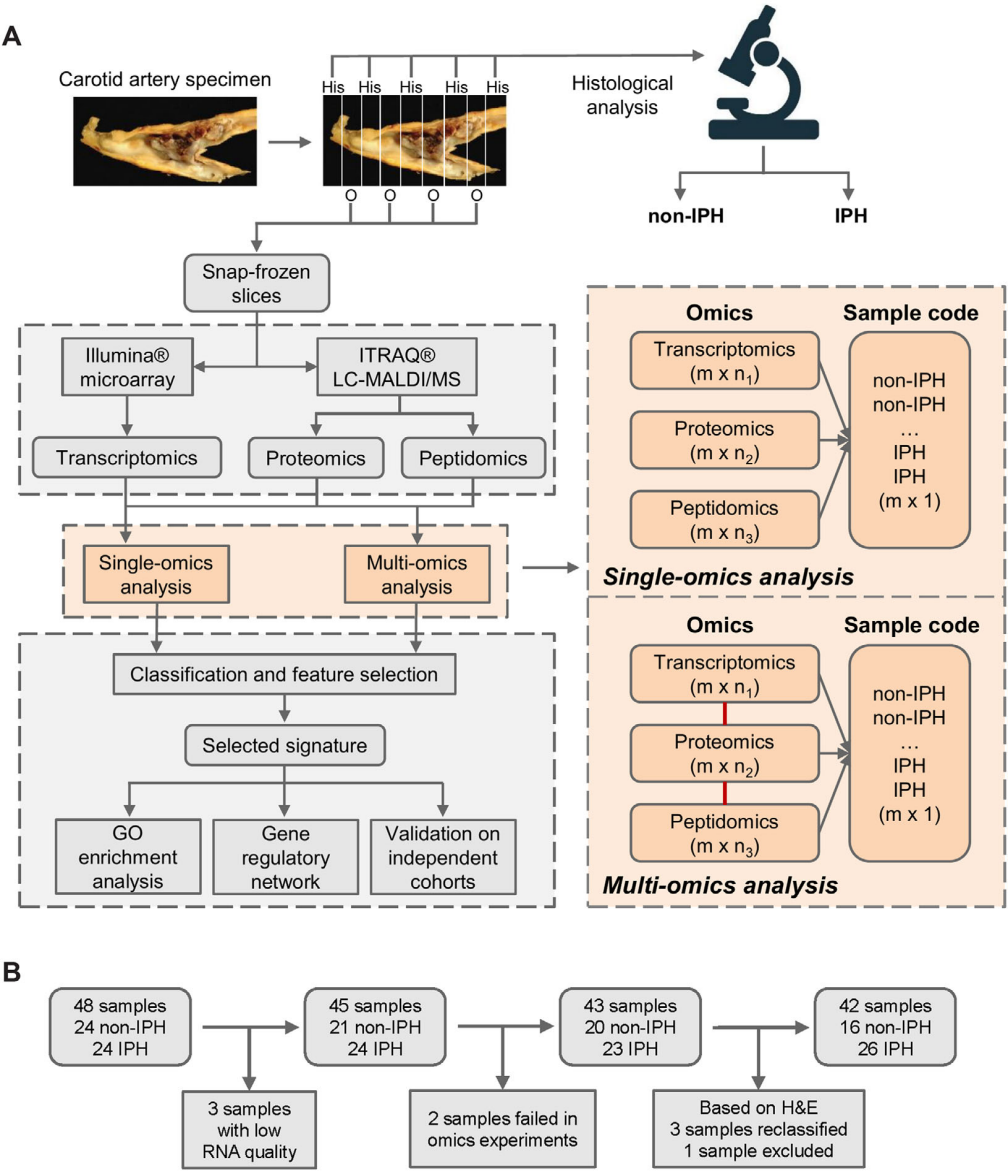
Workflow of carotid sample collection and data analysis. (A) Entire carotid endarterectomy specimen was cut in parallel 5‐mm thick slices, snap‐frozen in liquid nitrogen, and stored until use. Every second slice was sectioned. After H&E staining, sections were categorized using the criteria of Virmani et al.,^41^ and were further classified histologically based on the presence/absence of IPH, resulting in 16 non‐IPH and 26 IPH plaques for omics analysis. Workflow for single‐ and multiomics integrative sPLS‐DA analyses are shown in the orange dashed square. The integrative sPLS‐DA prediction model was obtained by connecting transcriptomics, proteomics, and peptidomics as an entirety; *m*: number of samples; n1, n2, n3: number of features. (B) Flow scheme of the MaasHPS cohort build‐up and the criteria for sample exclusion. In total, 42 samples (16 non‐IPH and 26 IPH plaques) were included in this study

Hematoxylin–eosin (H&E) stained plaque tissue was macroscopically preclassified for plaque stage before omics experiments. Segments were stratified into non‐IPH and IPH groups according to the absence or presence of IPH. For each CEA specimen and patient, two samples (one non‐IPH and one IPH) were collected for omics experiments (*n* = 24 at the start for each). However, one IPH and four non‐IPH samples did not pass the QC test and were excluded from analysis (see the following sections). Simultaneously with the microarray performance for the omics experiments, but prior to bioinformatic analysis, tissue was sectioned further for additional staining and computer‐aided quantitative measurement of plaque IPH. Based on this indepth reinspection, the three experienced pathologists (MJAPD, JCS, MJJG) agreed to remove one ambiguous “non‐IPH” sample, showing small but surface‐detached luminal fibrin clot, as this could represent either a surgery artefact or bona fide IPH. In three allegedly “non‐IPH” samples, quantitative morphometry detected minor IPH (0.43%, 0.40%, 0.33%, respectively, data not shown), which was overlooked in the preclassification. These three were recategorized as IPH after inspection by the pathologists. Due to the recategorization of samples from the non‐IPH to the IPH group, three pairs of the total 26 samples of the IPH group were from three patients, respectively. To explore the potential confounder effects of this adjustment, we performed hierarchical clustering of the samples based on the plaque traits (measured as described in Section 2.2). This analysis convincingly showed that there is no patient‐specific heterogeneity among the samples, as well as that plaque phenotype is dominant over sample origin (data not shown). The final cohort for this study therefore included transcriptomics, proteomics, and peptidomics for 16 non‐IPH and 26 IPH plaques (see Figure 1A,B for flow scheme of cohort build‐up). For detailed information on the patient cohort definition, see Table S1.

### 2.2 Morphometry and immunohistochemistry

All H&E slides were photographed at a 12.5× magnification and examined digitally using Leica Q500MC software. Total tissue, media, cap, necrotic core, hemorrhage, and luminal thrombus area were measured on H&E. Plaque size was calculated by subtracting the medial and luminal thrombus area from that of the total carotid tissue. Necrotic core and hemorrhage area were quantified relative to the plaque size. Cap thickness was measured at three to 15 positions throughout the entire cap region, at regular intervals. In addition, we measured the relative area of the following histological features: CD31^+^ endothelial cell content (% of total plaque area), CD3^+^ T‐cell content (% of total plaque area), CD68^+^ macrophage content (% of total plaque area), iNOS^+^ (M1) macrophage content (% of CD68^+^ macrophage area), Arg1^+^ (M2) macrophage content (% of CD68^+^ macrophage area), collagen content (% Sirius red of total plaque area), αSMA^+^ smooth muscle cell (SMC) content (% of total plaque area), αSMA^–^PDGFRα^+^ fibroblast‐like cell content (% of total plaque area), and calcification (% Alizarin red of total plaque area). These histological features are denoted as plaque traits, their distribution and statistical differences between paired non‐IPH and IPH plaques are shown in Figure S1. Moreover, a correlation heatmap was created to explore the relationships between the plaque traits (Figure S2).

For the MFAP4 immunohistochemistry for validation purpose, 4‐μm thick sections were cut from 35 plaques (13 non‐IPH, 22 IPH plaques) left available from the total 42 plaques. Deparaffinization and rehydration was performed using xylene and graded ethanol. Endogenous peroxidase was blocked with 0.3% H_2_O_2_ for 15 min, followed by heat‐induced antigen retrieval in a microwave for 10 min in a low pH target retrieval solution (K8005, DAKO, Agilent Technologies). After cooling down for 20 min, sections were incubated overnight at 4°C with polyclonal rabbit anti‐human MFAP4 antibody (1:1000, NBP2‐30439: Novus Biologicals). After washing in TBS, sections were incubated for 1 h at room temperature in Brightvision poly‐HRP anti‐rabbit IgG (ImmunoLogic, VWR KDPVR55 HRP). After washing in TBS, staining was developed using the chromogen 3,3′‐diaminobenzide (DAB, DAKO K3468) for 5 min, and sections were counterstained with Mayer's hematoxylin (VWR International B.V., Amsterdam, The Netherlands), dehydrated, mounted, and cover slipped. Sections were digitized using the Pannoramic 1000 scanner (3DHistech Ltd.) at 20× magnification. Morphometric analysis was done using the free software package QuPath (v0.2.3) to measure percentage area positivity/total area.

### 2.3 Sample preparation for transcriptomics and proteomics/peptidomics

Snap‐frozen omics segments were pulverized and 5–20 mg of material was subjected to transcriptomic, proteomic, and peptidomic analysis.

RNA was isolated by guanidium thiocyanate extraction, followed by further purification using the Nucleospin RNA II kit (Macherey‐Nagel GmbH & Co. KG). RNA concentration was measured using a Nanodrop ND‐1000 spectrophotometer (Nanodrop Technologies, Wilmington, DE, USA). RNA quality and integrity were determined on the Agilent 2100 Bioanalyzer (Agilent Technologies, Inc., Santa Clara, CA, USA). Pure RNA samples that had an RNA Integrity Number (RIN) greater than 6.0 and a sample purity A260/280 ratio greater than 1.8 were taken for transcriptomic analysis (45 out of 48 samples). One analysis for a non‐IPH plaque had failed, therefore in total four unqualified non‐IPH samples were excluded from the microarray experiment.

Proteomic analysis was carried out by utilizing the 8‐plex iTRAQ reagent for relative quantification. Reference sample used for relative quantification was created by pooling the redundant specimens derived from the equal portions of the samples for omics experiments. Pulverized tissue samples (5–20 mg) were homogenized in a Covaris E100 ultrasonic homogenizer using 400‐μl lysis buffer of 6 M guanidium hydrochloride, 1% Triton‐114, 50 mM triethylammonium bicarbonate (TEAB), 50 mM dithiothreitol (DTT), and protease inhibitor tablets (Roche, one‐fourth tablet in 10 ml buffer). The homogenate was centrifuged for 15 min at 4000 × *g* to remove insoluble debris. DTT content of the homogenate was raised to 100 mM and protein reduction was completed through incubation at 70°C for an hour. Alkylation was completed by adding 0.5 M iodoacetamide and followed by a 30‐min incubation at room temperature. The excess alkylating reagent was quenched by addition of 12 μl of 1 M DTT. The protein content of the homogenate was recovered on a poros R1 column using a Vision Chromatography Station (Applied Biosystems). Proteins were eluted with 70% isopropanol/30% formic acid solvent mixture and dried down in a Speedvac.

### 2.4 Omics data collection

Biotinylated cRNA was prepared using the Illumina TotalPrep RNA Amplification Kit (Ambion, Inc., Austin, TX, USA) according to the manufacturer's specifications starting with 100 ng total RNA. Per sample 750 ng of cRNA was used for hybridization. Hybridization and washing were performed according to the Illumina standard assay procedure. Scanning was performed on the Illumina BeadStation 500 (Illumina, Inc., San Diego, CA, USA), while image analysis and extraction of raw expression data were done using Illumina Beadstudio v3 Gene Expression software with default settings (no background subtraction and no normalization). Transcripts were measured by Illumina HumanRef‐8 v2.0 expression BeadChip.

Proteins were resuspended in 2 M freshly prepared urea, 1 M TEAB, 1% n‐octyl‐glucoside buffer and digested with trypsin added at 1:20 w/w ratio to the sample, for 4 h at 37°C. Digestion was stopped by increasing the temperature to 95°C for 5 min. Digested samples were labeled by the 8‐plex iTRAQ reagents following the manufacturer's protocols (Applied Biosystems) using a sample digest quantity that represents approximately 40‐μg protein content. Samples were labeled with the reagents yielding the *m*/*z* 114, 115, 116, 118, 119, 121 reporter fragments in the MS/MS scans. Reference samples were labeled with the 113 and 117 reagents. iTRAQ labeled reactions were quenched by the addition of 1 M ammonium bicarbonate.

Eight samples (three non‐IPH, three IPH, and two replicates for QC and reference) constituting an iTRAQ mix were combined, desalted, and fractionated by strong cation exchange (SCX) chromatography using an Agilent 1200 instrument. Eight SCX fractions were injected for HPLC, and resolved over a 90‐min gradient of 5% solvent B (10% H2O/90% ACN/0.1% TFA) to 38% B (solvent A: 95% H2O/5% ACN/0.1% TFA). The elution volume was collected onto a plate for mass spectrometric analysis by matrix‐assisted laser desorption ionization (MALDI) as 10‐s intervals, using 10 mg/ml of alpha‐cyano‐4‐hydroxycinnamic acid in 50%–50% acetonitrile–water as the matrix. Each HPLC run was represented as a 500‐spot array on the MALDI plate. These plates were analyzed on an AB4800 mass spectrometer (MDS/SCIEX, Concord, ON, Canada).

Peptide quantification was carried out by calculating the average ion intensity ratios relative to the *m*/*z* 113 and 117 peaks. Protein ratios were determined as the medians of all peptide ratios matching to the same protein. Peptide sequences were identified from MS/MS fragmentation spectra using the Mascot search engine (Matrix Science, UK) and the SwissProt database. The whole proteomic/peptidomic analysis was carried out following the same protocols as in a previous plasma proteomics study for cardiovascular disease.^26^ Experimental details and parameters used in this analysis can be found from the paper given above. Once all the study samples were analyzed, the complete peptide set was remapped to a minimum protein set, whereafter proteins and peptides identified were mapped to genes or gene families. The list of protein and peptide entries used in the multiomics integration analysis can be found in Table S2.

The corresponding microarray profile of one IPH sample with failed MS measurement (due to technical reasons) was removed. The final MaasHPS cohort therefore included transcriptomics for 16 non‐IPH and 26 IPH plaques, with successful proteomics and peptidomics profiling from the same samples.

### 2.5 Data preprocessing

A total of 22,184 human transcripts and variants as defined by the NCBI Reference Sequence (RefSeq) were analyzed. Transcriptomic data were analyzed using the R Bioconductor lumi package (v2.38.0). First, we performed a variance stabilizing transformation, which is incorporated in the lumi package. Then, the robust spline normalization (RSN) algorithm in the lumi package was applied to normalize the data. As low‐variance genes and noise expression will not only reduce the effectiveness of subsequent clustering and machine learning but also slow down computations, we selected the top 10,000 most variable genes from a total pool of 17,759 unique detectable genes, for further analysis.

Measured values of the abundance of 1330 proteins and 4736 peptides were normalized using a procedure based on Vandesompele et al.^27^ Intrinsic to the analysis methodology, datasets were showing a considerable rate of missing values (26.45% and 38.94%, respectively). To reduce the noise and bias affected by features with sub‐ and peri‐threshold abundance, we discarded all features with ≥50% missing values. For the remaining features, missing values were imputed by *k*‐nearest neighbors (*k*‐NN) imputation (*k* = 7) in an unsupervised manner, as this proved superior to other methods in a pilot analysis.^28^

Finally, 10,000 genes, 943 proteins, and 2637 peptides were detectable in the 42 samples (16 non‐IPH vs. 26 IPH) and were used for further analysis. Genes, proteins, and peptides were mapped based on the HUGO Gene Nomenclature Committee (HGNC) symbols.

### 2.6 Single‐ and multiomics data analysis

For single‐omics analysis of the transcriptomic, proteomic, and peptidomic data, we deployed sparse partial least squares‐discriminant analysis (sPLS‐DA, or single sPLS‐DA),^29^, ^30^ a multivariate methodology which allows sample classification and feature selection by projecting the data into a lower dimensional space in a supervised manner. While applying this method on omics datasets, which typically have a high‐dimensional feature space with limited sample size, this algorithm implements LASSO^31^ to limit the number of features used in the model so as to reduce the curse of dimensionality. To enable classification based on multiblock datasets, derived from transcriptomic, proteomic, and peptidomic analysis of plaques, we deployed integrative sPLS‐DA using DIABLO provided in the mixOmics R package (http://mixomics.org/, v6.8.5),^25^ a powerful package shown to extract biologically relevant signatures from multiple omics data by maximizing the sum of the covariance between all pairs of latent components from each dataset and projecting the different omics data into a common space.^32^ Compared with other multiomics machine learning algorithms, this method has several advantages. First, it allows full integration of multiomics data by extracting common information from different omics layers for classification. Second, it enables model‐embedded feature selection, which is a great help for identifying disease‐specific novel biomarkers. Third, this method provides clear and interpretable visualization, facilitating downstream exploration and interpretation by biologists. The inherent characteristics^33^ and the successful applications of sPLS‐DA on several microarray data^34^, ^35^ suggest the suitability of using this method in our study.

### 2.7 Parameter optimization

SPLS‐DA requires extensive optimization of both the number of components and the number of features for constructing each component. In brief, the number of testing components was set from one to six, as in most cases, the prediction performance deteriorates rapidly when the number of the components increase above five. Then, the number of features to be tested for constructing the sPLS‐DA component was configured. For single sPLS‐DA on transcriptomics, proteomics, and peptidomics, a range of features from three to 300 (from three to 30 in steps of three, from 30 to 60 in steps of six, from 60 to 150 in the step of 15, and from 150 to 300 in the step of 30) were set for each component. For integrative sPLS‐DA, to achieve the comparable number of selected features, the range of testing features here for each component and each omics was set from 10 to 100 (from 10 to 50 in steps of five, from 50 to 100 in steps of 10, in total 14 candidates per component per omics).

Specifically, for integrative sPLS‐DA, a design matrix representing how close the datasets should be connected to each other needs to be configured. According to Singh et al.’s suggestion,^36^ we configured the design matrix C as the correlation between omics datasets as 0.1, and the correlation between omics dataset and outcome dummy matrix as 1.

Optimal parameters can be derived in one step from the model with the best classification in cross‐validation. A series of single and integrative sPLS‐DA prediction models were obtained from each combination of these parameter settings, and their performances were tested under stratified five‐fold cross‐validation with 1000 random repeats. Considering the unbalance of the sample phenotypes, to fairly reflect the classification performance of training models, in this tuning phase we used balanced error rate (BER), which takes the average of the errors on each class to evaluate the classification error. The optimal parameters can be concluded from Figure S3A (for integrative sPLS‐DA, data not shown) and are listed in Table S3. The performance of the single and integrative sPLS‐DA models with the given optimal parameters was evaluated by accuracy and area under the curve of receiver operating characteristic (AUC) under stratified five‐fold cross‐validation with 10,000 random repeats (Figure S3B).

### 2.8 Feature selection

Inspired by the method used in a previous multiomics study,^32^ to ensure that selected features had broader significance beyond the single best prediction model, we performed stratified sampling with replacement generating 10,000 incomplete copies of the full dataset; each copy covered 80% of the original data, and phenotype distribution was identical to that of the original dataset. We then ran single‐ and multiomics analysis on each copy, selecting the overall highest ranking features by aggregating the loading weight value (i.e., the importance to the model) of each feature from each copy to obtain the final feature subset. The optimal parameters (Table S3) were used to determine the number of the selected features.

To simplify the downstream analysis, we assembled gene, peptide, and protein identifiers of selected features into a final feature set, unifying the identifiers into the corresponding gene symbol and excluding duplications. The final selections used for overrepresentation analysis, transcriptional regulatory analysis, and validation entailed 291 and 283 gene features, for single and integrative sPLS‐DA, respectively. The full lists of the selected features are presented in Table S4, sorted by the overall feature importance from high to low.

### 2.9 Bioinformatic analyses

#### 2.9.1 Differential gene expression analysis

We used the function lmFit() provided in the limma R package (v3.42.2) for differential expression analysis on preprocessed transcriptomics between IPH and non‐IPH groups. Positive log2 fold change (Log2FC) values indicate genes have higher expression in IPH group, and vice versa. Results of differential expression analysis for the 17,759 unique detectable genes can be found in Table S5.

#### 2.9.2 Gene set overrepresentation analysis (GSOA)

We performed GSOA using the R packages clusterProfiler^37^ (v3.12.0) and ReactomePA^38^ (v1.28.0) identifying biological functions of the selected gene sets. Three categories of GSOA datasets were queried: Gene Ontology (Biological Process GOBP, Molecular Function GOMF, and Cellular Component GOCC), Reactome Pathway Database (REACTOME), and Kyoto Encyclopedia of Genes and Genomes (KEGG). All genes covered by this Illumina microarray platform and all detectable proteins/peptides in the MS experiments were used as the background. Benjamini–Hochberg adjusted *p*‐values were calculated as a cutoff to avoid presenting the false discovery of significant terms. The full list of GSOA results for both single and integrative sPLS‐DA can be found in Table S6.

#### 2.9.3 Transcriptional regulation analysis

iRegulon^39^ (v1.3), a Cytoscape plugin, was used to map transcription factors (TFs) driving a gene network. The selected features by integrative sPLS‐DA were set as input in iRegulon for TF discovery. By setting the default parameters, we have a broad search space including 9713 position weight matrices (PWMs) and 1120 ChIP‐seq tracks. The putative regulatory region was set to 20 kb centered around transcription start sites (TSS). Top five TFs were ranked by normalized enrichment score (NES) and visualized as a transcriptional regulatory network.

### 2.10 Validation cohorts and approach

The predictive power of genes from the multiomics signature was validated in the following independent human carotid endarterectomy cohorts on microarray gene expression: GSE28829^40^ (*n* = 29; 13 early and 16 advanced plaques) and GSE43292^4^ (*n* = 64; 32 plaque‐free artery segments and 32 atheromatous plaques).

The prediction performance of the gene lists obtained from integrative sPLS‐DA components 1–4 (MULTI 1–4, *n* = 283) and single sPLS‐DA components 1–2 (SINGLE, *n* = 291, for peptidomics only component 1) were tested by logistic regression (solver = “lbfgs”), support vector machine (SVM, kernel = “rbf,” gamma = “auto”) and decision tree (default settings) with stratified five‐folds cross‐validation and 1000 random repeats provided in the scikit‐learn machine learning package (v0.24.1) in Python. As the integrative sPLS‐DA component 1 (MULTI 1, *n* = 22) was dominant in our model, we also compared the performance of the MULTI 1 signature with that of MULTI 1–4 signature from the complete four‐component model. Classification performance using full data (FULL) was evaluated as a baseline. Predictive performance was evaluated by two measures: AUC and accuracy.

### 2.11 BiKE verification

BiKE cohort patient inclusion for CEA surgery and enrolment in the biobank, clinical data, sample processing and large‐scale datasets have been described in details previously.^7^, ^18^ In this study, the classification performance of MULTI 1 was also validated on transcriptomics (*n* = 137; 127 carotid plaques and 10 normal arteries) and proteomics (*n* = 36; 18 carotid plaques and 18 adjacent, healthy, matched segments) from the BiKE cohort. Within the BiKE, two subcohorts were defined. For the first, we compared non‐atherosclerotic tissues (normal; *n* = 10 for transcriptomics, *n* = 18 for proteomics) versus atherosclerotic arteries (plaques; *n* = 127 for transcriptomics, *n* = 18 for proteomics), based on their morphological and histological characteristics (HIST). For the second, carotid plaque tissues were classified as asymptomatic (*n* = 40 for transcriptomics, *n* = 9 for proteomics) versus symptomatic (*n* = 87 for transcriptomics, *n* = 9 for proteomics) based on the patient's clinical presentation (CLIN). For both subcohorts, we tested the classification performance of the identified multiomics signature MULTI 1 on transcriptomics and proteomics, respectively, using logistic regression with the same setting mentioned above for GSE28829 and GSE43292. Gene set enrichment analysis (GSEA) was performed on the signature MULTI 1 against the genes and proteins from BiKE transcriptomics and proteomics, with the genes/proteins ranked by log2 fold change based on the comparisons of HIST and CLIN.

### 2.12 Statistical analysis

Component–component and component–trait associations were measured by Pearson's correlation coefficients with *p*‐values. Classification performances are presented as the mean ± standard deviation (SD). Distribution of plaque traits and IHC‐based MFAP4 quantification for paired plaques are presented as box and whisker plots with the first quartile, median, third quartile, and the largest or smallest values within 1.5 times the interquartile range above the third quartile or below the first quartile, respectively. Statistical significance between groups was evaluated using two‐tailed Wilcoxon rank‐sum test (for nonnormally distributed data) or Student's *t*‐test (for normally distributed data). For the MaasHPS data, paired statistical testing was performed. Shapiro–Wilk test was used for the normality test. Statistical significance between the classification performance was analyzed by Student's *t*‐test. Significance level is denoted by *p‐value < .05, **p‐value < .01, ***p‐value < .001, ****p‐value < .0001. Statistical analyses were performed in R (v3.6.3).

## 3 RESULTS

In this study, we deeply phenotyped low‐ versus high‐risk human carotid artery atherosclerotic lesion segments, as defined by the absence or presence of IPH, building an integrated gene/protein classification model for IPH. For this, we interrogated a carotid endarterectomy cohort (MaasHPS) by transcriptomic (microarray), proteomic, and peptidomic analysis (both LC‐MALDI‐MS/MS). Flanking sections were taken for detailed histological examination of cellular and acellular plaque composition and progression stage^41^ (Figure S1), resulting in 42 CEA samples (16 non‐IPH vs. 26 IPH, Table S1) from 24 patients (see Materials and methods section and Figure 1A,B for cohort build‐up scheme). After preprocessing, a total of 10,000 genes, 943 proteins, and 2637 peptides were analyzed according to the workflow described in Figure 1A. For clarity sake, mRNAs/genes, proteins, and peptides from omics data are termed features, and a set of features is termed signature.

### 3.1 Single‐omics sPLS‐DA

**FIGURE 2:**
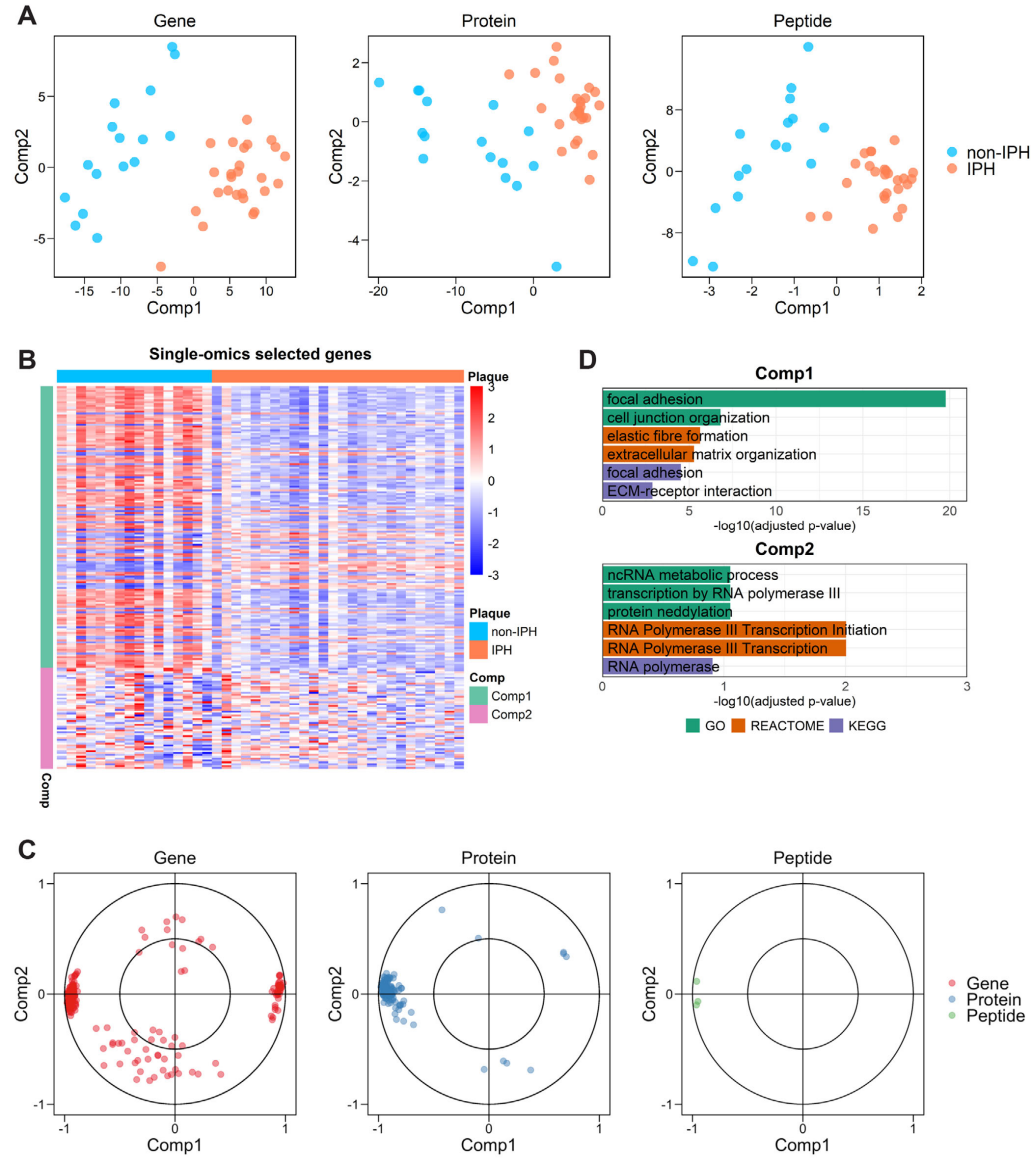
Single‐omics sPLS‐DA on transcriptomics, proteomics, and peptidomics. (A) Two‐component sample distribution (16 non‐IPH and 26 IPH) for single‐omics sPLS‐DA on plaque transcriptomics (gene), proteomics (protein), and peptidomics (peptide), respectively. For visualization purpose, two components were included in the plot for peptidomics. (B) Heatmap shows expression level of the selected genes (comp1, *n* = 150; comp2, *n* = 54) from transcriptomics. Expression values were *z*‐normalized per row. (C) Selected features by single sPLS‐DA on the three omics are plotted, respectively (*n* = 204 for genes; *n* = 111 for proteins; *n* = 3 for peptides), according to their Pearson's correlation coefficient to the components 1 and 2. (D) GSOA on standardized gene set of single sPLS‐DA. For each component, six highly ranked overrepresented terms are selectively shown

First, we built single sPLS‐DA models based on the transcriptomic, proteomic, and peptidomic datasets, separately. The optimal parameters for model building can be found in Table S3 and Figure S3A. As is evident from Figure 2A, all three models were able to segregate IPH from non‐IPH plaque at high predictive performance, with accuracies of approximately 0.9 and AUCs of >0.95 (Figure S3B); in fact, nearly 90% of samples could be correctly classified by all three omics datasets separately. Next, we constructed heatmaps for the three omics layers, respectively, depicting the differential expression of signature members by IPH versus non‐IPH plaque (Figure 2B and Figure S4A). Component 1 gene, protein, and peptide signatures showed strong differential expression between plaques with or without IPH. This is in support of the discriminative power of component 1 (Figure 2A), and suggests that the first component is the dominant factor in the prediction model.

Next, we mapped the distribution of the selected feature sets (Figure 2C), based on the Pearson's correlation coefficient between the latent components (i.e., components 1 and 2) and the selected features.^42^ For both components, selected features were distributed differently, centering toward the extreme ends of component 1, and being more diffusely distributed for component 2. Thus, unlike component 2, component 1 features showed pronounced mutual interaction, suggesting involvement in a coordinated biological process. GSOA of the selected signatures for the first, decisive component revealed a clear overrepresentation of fibroblast and extracellular matrix‐associated biological processes (Figure 2D, Table S6). In contrast, but expected based on the diffuse distribution in Figure 2B,C, component 2 did not offer deeper insights into hemorrhage relevant processes. Of note, even for component 1, the level of overrepresentation was rather low, precluding firm conclusions based on single sPLS‐DA only.

### 3.2 Multiomics sPLS‐DA

**FIGURE 3:**
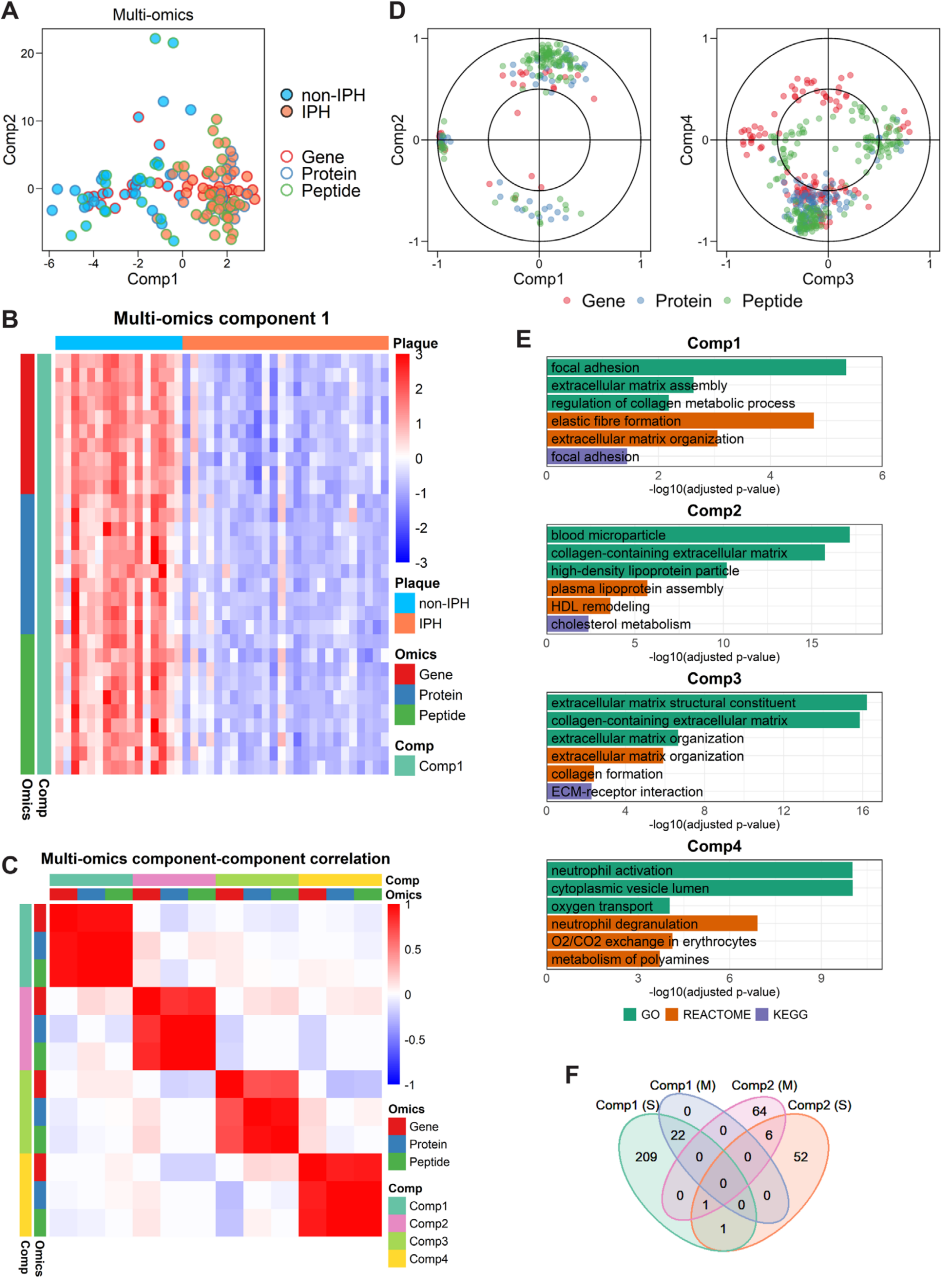
Multiomics sPLS‐DA on transcriptomics, proteomics, and peptidomics. (A) Two‐component sample distribution for multiomics sPLS‐DA on plaque transcriptomics (gene), proteomics (protein), and peptidomics (peptide). Non‐IPH and IPH plaques are denoted in skyblue and coral, samples from different omics layer (16 non‐IPH and 26 IPH for each omics layer) are denoted by red, blue, and green borders. Considering the high consistency between the components of the three omics layers in the multiomics model (see C), three sample distributions for different data layers were combined into one plot. (B) Heatmap shows expression/abundance level of the selected genes (*n* = 10), proteins (*n* = 10), and peptides (*n* = 10) for the first component of multiomics analysis. Expression values were *z*‐normalized per row. (C) Heatmap shows correlations between components from the multiomics model. (D) Distribution of the selected genes, proteins, and peptides on their corresponding components of multiomics sPLS‐DA. Elements are plotted according to their Pearson's correlation coefficients to the components 1 and 2 (*n* = 30 for genes; *n* = 50 for proteins; *n* = 110 for peptides), or 3 and 4 (*n* = 125 for genes; *n* = 100 for proteins; *n* = 170 for peptides). (E) GSOA on standardized gene set of multiomics sPLS‐DA. For each component, six highly ranked overrepresented terms are selectively shown. (F) Overlapping between standardized gene subset of single‐omics (S) components 1 and 2, and multiomics (M) components 1 and 2

In order to establish a more comprehensive and robust model of IPH, we sought to analyze plaque transcriptomic, proteomic, and peptidomic data in an integrated manner by integrative sPLS‐DA using the optimized parameters in Table S3. The resulting prediction model provided excellent discrimination of non‐IPH versus IPH plaque (Figure 3A), and performed at least equally good as all of the single‐omics models, in terms of accuracy and AUC (Figure S3B). The features of the first, but not second to fourth components, showed overt differential expression between non‐IPH and IPH plaque (Figure 3B and Figure S4B), regardless of their origin (i.e., gene, protein, peptide). Furthermore, the selected proteins and peptides in Figure 3B showed considerable overlap (12 out of 20, Table S4). Also, for each of the other three components, part of the selected proteins and peptides are overlapping (Table S4), implying the contribution of the proteomics and peptidomics to the multiomics model are to some extent similar but not identical. Cross‐component correlation analysis (Figure 3C) confirmed the strong mutual correlation of component members from the three omics layers, whereas cross‐interaction across the components was very weak, underpinning the high level of independence of the four components of our model.

**FIGURE 4:**
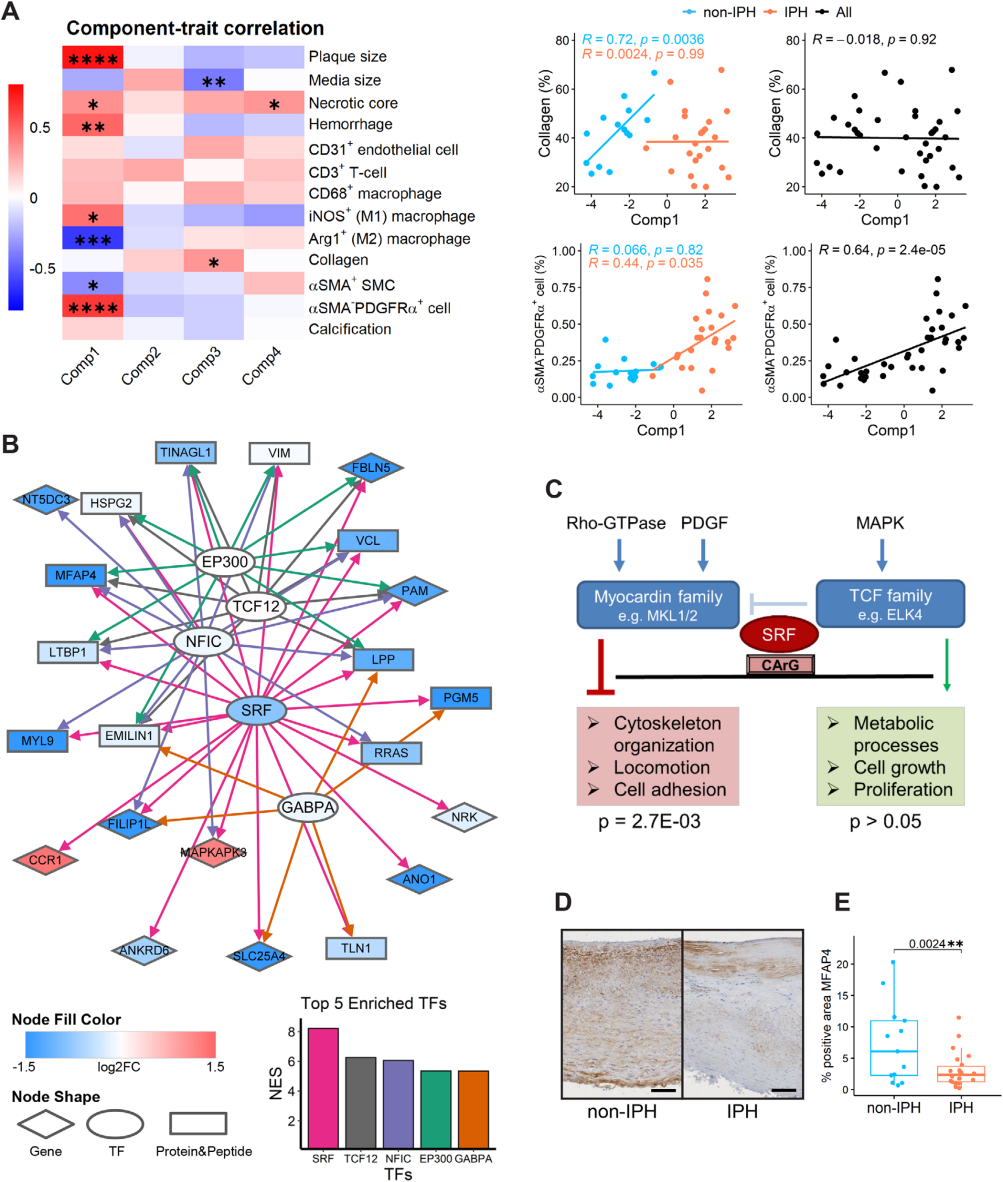
Biological extension of the results of the multiomics analysis. (A) Correlation‐based heatmap shows correlations and corresponding *p*‐values (denoted as asterisk) between multiomics components 1–4 (derived from the transcriptomic block) and plaque traits. Specifically, correlations of components with collagen content and αSMA^–^PDGFRα^+^ fibroblast‐like cell content are shown as scatter plots (non‐IPH, *n* = 16; IPH, *n* = 26; all plaques, *n* = 42). (B) Transcription factors (TFs) targeting the elements from the first component of multiomics sPLS‐DA‐based set of genes/proteins/peptides were extracted by iRegulon with default settings. Likely interactions of TFs with their downstream targets as obtained from iRegulon are indicated by directed lines with different color per TF. Genes, proteins and peptides, and TFs are depicted in different shapes; gene‐level differential expression (log2 fold change) is shown by green‐red gradient. The top five enriched TFs for multiomics component 1 ranked by normalized enrichment score (NES) are shown below, with the same colors as the directed lines in the network. (C) SRF network operating in plaques primarily steers the MKL1/2, not the ELF4 coregulated pathway. The *p*‐values were calculated by hypergeometric test. (D) Representative pictures of IHC for MFAP4 (brown area) in non‐IPH and IPH plaques. Scale bar = 100 μm; 100× magnification. (E) Differences of IHC‐based MFAP4 positive area (%) between paired non‐IPH and IPH plaques (both *n* = 13)

To add biological meaning to the prediction model, we set out to identify cofunctional gene/protein/peptide clusters, correlating with each of the four components of the integrative model (Figure 3D). Several clusters were observed, suggesting a very strong mutual correlation between genes, proteins, and peptides. This points to coordinate function and/or regulation of the cluster members. GSOA of the gene, protein, and peptide members underlying the integrated classification model showed a clear cardiovascular signature (Figure , Table S6), with marked overrepresentation of extracellular matrix organization (components 1 and 3), lipid metabolism (component 2), and immune response terms (component 4). Interestingly, components 1 and 3 showed considerable functional overlap, despite their low interdependence. Of note, the first component of the multiomics model was very comparable to that of the single‐omics models, and showed a significant level of overlap (Figure 3F). To validate the GSOA findings, we correlated the four components with several important plaque traits, such as IPH, fibrosis, and macrophage presence (Figure 4A, Table S7). The dominant first component was highly correlated with plaque size (correlation = 0.74, *p* = 1.8E‐08) and hemorrhaged plaque area (correlation = 0.48, *p* = 1.4E‐03), as expected, as well as with αSMA^–^PDGFRα^+^ fibroblast‐like cells (correlation = 0.64, *p* = 2.4E‐05), Arg1^+^ healing macrophage phenotype (correlation = −0.62, *p* = 2.2E‐04), and collagen content of non‐IPH plaque (correlation = 0.72, *p* = 3.6E‐03), concordant with the GSOA findings (Figure 3E). Indeed, plaque size, hemorrhage area, collagen content, and αSMA^–^PDGFRα^+^ cell content were significantly correlated with each other (Figure S2), possibly explaining the correlations between component 1 and these plaque traits (Figure 4A).

Given the functionally related signature members in integrative sPLS‐DA component 1, suggesting a shared regulatory basis, we performed regulatory analysis by iRegulon to identify TFs driving this signature set, and built a regulatory network of the IPH signature encompassing mRNAs, protein, peptides, and in silico‐identified TFs. As can be seen from Figure 4B, all the 22 nonredundant members of component 1 were targeted by the top five enriched TFs. MRNA of the highest ranked TF, serum response factor (SRF), was significantly and sharply downregulated in hemorrhaged plaque regions, mirroring the effects seen on most of the SRF connected network members. SRF signaling is tightly dependent on its cofactor, where myocardin family members drive locomotion and cell adhesion activities, while T‐cell factors promote proliferation. Hypergeometric testing using the reference dataset of Xie's study^43^ showed a clear overrepresentation of SRF‐targeting network members in megakaryoblastic leukemia‐1/2 (MKL1/2) regulated SRF‐dependent genes (*p*‐value = 2.7E‐03), but not in ETS‐domain protein 4 (ELF4) regulated SRF‐dependent genes (Figure 4C). This points to the former as the dominant signaling pathway in non‐IPH plaque, promoting actin cytoskeleton organization, locomotion, and cell adhesion regulated by SRF in plaque.

One of the SRF‐regulated features with the strongest downregulation in IPH compared to non‐IPH, at gene expression level (Figure 4B), was microfibril‐associated glycoprotein 4 (MFAP4), which has been described to localize in vascular wall extracellular matrix fibers and to be involved in neointima formation.^44^ Immunohistochemical validation of MFAP4 presence in plaque sections from the same cohort (MaasHPS) indeed confirmed its reduced abundance in hemorrhaged plaques (Figure 4D,E). We also examined the MFAP4 gene expression and protein abundance in BiKE cohort, which showed a downregulation in atherosclerotic arteries compared to normal or adjacent tissues, as well as in symptomatic patients compared to asymptomatic patients (Figure S5A). Moreover, based on GSEA, we observed significant negative NES of the SRF‐regulated network members in BiKE cohort for the comparisons of HIST and CLIN, suggesting the general downregulation of the network members in carotid plaques compared with normal or adjacent tissues, and in symptomatic patients compared with asymptomatic patients (Figure S5B), which is in line with the expression in Figure 4B.

### 3.3 Independent validation of findings from multiomics analyses

Finally, we assessed whether the IPH signature was able to discriminate low‐ from high‐risk plaques in two independent mRNA cohorts in the public domain: GSE28829^40^ (*n* = 29; mRNA; early vs. advanced plaque) and GSE43292^4^ (*n* = 64; mRNA; intact arterial tissue vs. atheroma). Although the cohort setup differs from our cohort, carotid advanced plaque (GSE28829) and atheroma (GSE43292), both largely represent unstable plaque phenotypes (thin cap fibroatheroma and beyond). The main difference is in the control tissue with even lower risk than our nonhemorrhaged stable plaque: that is, early‐stage lesion (low risk) and nonlesioned arterial tissue, in the two validation cohorts, respectively, versus nonhemorrhaged stable plaque in our study, but we argued that our signature may still be valid for these broader disease trajectories.

**FIGURE 5:**
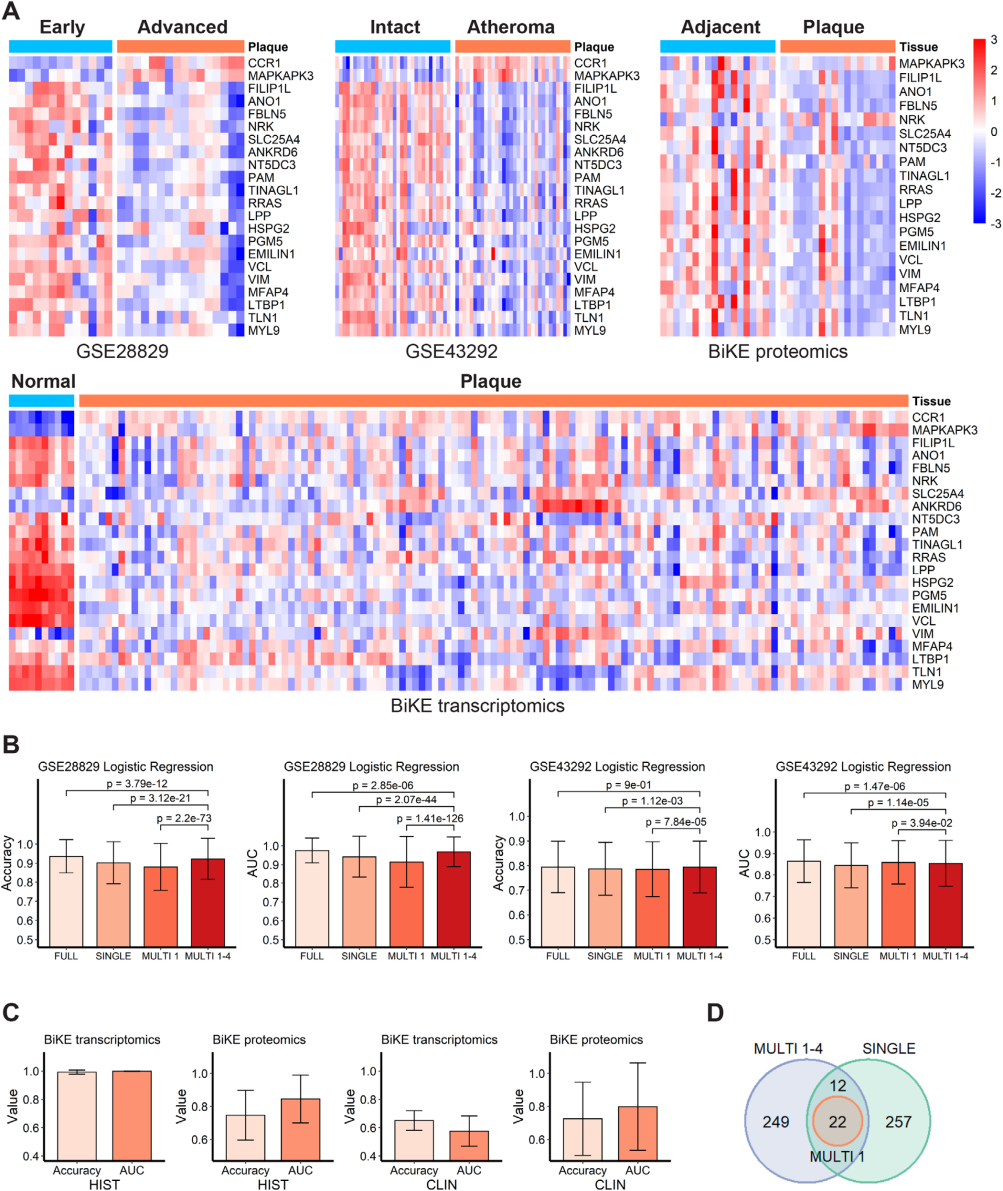
Validation of selected gene sets using independent cohorts. (A) Heatmaps shows expression/abundance pattern of the selected genes/proteins from component 1 by integrative sPLS‐DA analysis (MULTI 1) in validation cohorts. For GSE28829, early (*n* = 13) versus advanced (*n* = 16) plaques; for GSE43292, intact arteries (*n* = 32) versus atheromata (*n* = 32); for BiKE proteomics, adjacent tissues (*n* = 18) versus plaques (*n* = 18); for BiKE transcriptomics, normal tissues (*n* = 10) versus plaques (*n* = 127). Expression values were *z*‐normalized per row. (B) Classification performance based on full data (FULL), single‐omics (SINGLE), multiomics component 1 (MULTI 1), and multiomics components 1–4 (MULTI 1–4) feature subsets on two independent cohorts GSE28829 and GSE43292. (C) Classification performance of MULTI 1 feature subsets on BiKE cohort in distinguishing normal (*n* = 10 for transcriptomics; *n* = 18 for proteomics) versus plaque (*n* = 127 for transcriptomics; *n* = 18 for proteomics) based on histological characteristics (HIST), or asymptomatic (*n* = 40 for transcriptomics; *n* = 9 for proteomics) versus symptomatic (*n* = 87 for transcriptomics; *n* = 9 for proteomics) based on clinical events (CLIN). For B and C, results were measured by accuracy and AUC using logistic regression, with stratified five‐fold cross‐validation and 1000 random repeats. Results are presented as mean ± SD. (D) Overlapping between feature sets SINGLE, MULTI 1, and MULTI 1–4

Indeed, in both cohorts, the signature genes from integrative sPLS‐DA component 1 (MULTI 1) were differentially expressed between control versus diseased artery (Figure 5A), with general downregulation in advanced plaque. Both the single‐omics (SINGLE) and the multiomics (MULTI 1, MULTI 1–4) signatures performed very well for the logistic regression and SVM classifiers, with AUCs of 0.95 in advanced versus early (GSE28829, Figure 5B and Figure S6A). Of note, the first component of the multiomics model (MULTI 1) by itself already performed surprisingly well in stratifying the two plaque phenotypes for GSE28829, albeit not as good as the complete four‐component gene set (MULTI 1–4, Figure 5B and Figure S6A,B). Apparently, the first component is dominant in stratifying the validation cohorts, with a complementary contribution of components 2–4. Validation in GSE43292 did not show major differences in predictive power of the SINGLE, MULTI 1, and MULTI 1–4 gene sets, with overall high performance (AUCs >0.8) for all gene sets tested by logistic regression and SVM (Figure 5B and Figure S6A). Decision tree classifiers performed less impressive than the other two classifiers (Figure S6B). Here, the MULTI 1–4 gene set appeared to be superior to the other models (Figure S6B), possibly owing to the structure of the decision tree classifier, in which the splitting nodes can incorporate the independent features from distinct multiomics components.

Finally, we validated performance of the MULTI 1 signature in the BiKE cohort study, which includes both high‐powered transcriptomics (*n* = 127) and proteomics datasets (*n* = 36) from CEA tissue. The MULTI 1 feature set performed well on both the transcriptomics and proteomics in segregating advanced plaque from healthy carotid tissue (histological, HIST; see Figure 5C). Interestingly, while both involved hemorrhaged plaques, our signature was even able to stratify plaques from symptomatic versus asymptomatic patients based on their proteomics at a reasonable classification performance (AUC = 0.80), and to a lesser extent transcriptional profile (AUC = 0.57; clinical, CLIN; see Figure 5C). Indeed, all the MULTI 1 members overlap with MULTI 1–4 and SINGLE (Figure 5D), suggesting the MULTI 1 signature derived from the dominant component of the multiomics model is essential for plaque classification.

## 4 DISCUSSION

Elucidation of critical processes in the transition of low‐risk into high‐risk rupture‐prone plaque in humans will pave the way for early diagnosis of, and targeted intervention in atherosclerosis‐related cardiovascular diseases. Here we have deployed integrative analysis of transcriptomic, proteomic, and peptidomic expression/abundance profiles of human carotid artery plaque to build a multitethered prediction model for distinguishing low‐ from high‐risk carotid artery lesions. The significance of this model was corroborated in multiple independent plaque cohort studies. Finally, based on this model, we constructed an SRF‐driven regulatory gene/protein network overrepresented in extracellular matrix remodeling and interaction terms, which could serve as a starting point for the design of a targeted intervention.

Single‐omics studies on human atherosclerotic plaque by us (this study, Goossens et al., Eijgelaar et al.)^22^, ^45^ and others^4^, ^5^, ^6^, ^8^ have already shown the power of these high‐throughput approaches for biomarker discovery and mechanistic studies. For instance, Ayari and Bricca have identified the CD163/HO‐1 axis to be associated with iron–heme homeostasis in atherosclerotic plaque, using differential expression analysis on microarray data extracted from 68 carotid atheroma specimens.^4^ However, single‐omics approaches intrinsically fail to capture the complexity of biological systems as a whole, providing useful but incomplete information.^46^, ^47^ Moreover, genomic and peptide/protein expression/abundance in plaque were seen to show poor mutual correlation.^15^ Consequently, genomics findings are not directly translatable to (pre)clinical application, while protein‐based models generally are rather sparse, biased toward high‐abundance proteins and lacking their regulatory context. This combined with the observation that both domains display distinct but complementary correlations with relevant clinical traits pleads in favor of integrative multiomics analysis on atherosclerosis to have the best of both worlds, producing a robust, translatable model of disease progression.

This study illustrates the benefits of truly integrative omics analysis in terms of accuracy, visibility, and model interpretability. Our multiomics prediction model achieved similar, if not higher, accuracy in stage classification as a single‐omics model. The prediction power of the selected features was successfully validated on several independent cohorts comparing healthy artery or early (stable) plaque versus advanced (unstable) plaque or atheroma. Even, the selected features performed reasonably well in stratifying plaques from symptomatic versus asymptomatic patients at protein level (BiKE proteomics), although plaques in both groups were having an advanced (unstable) phenotype. However, the true merit of integrative analysis is in the robustness of the model, its biological significance, and the direct clinical translatability of network‐contained proteins. Significances of overrepresented terms in the multiomics signature outperformed those of the single‐omics signature, due to the strong mutual interaction of signature members per component. Interestingly, while the dominant component of the single‐ and multiomics models showed considerable overlap, the auxiliary second to fourth components of the single‐ and multiomics models differed substantially, with those of the latter being much more coherent and biologically meaningful. The dominant component in the multiomics model was overrepresented in collagen metabolism and macrophage inflammation terms, reflecting peri‐rupture modeling processes. The auxiliary components alluded to additional CVD relevant terms, such as lipid and lipoprotein metabolism, neutrophil responses and, again, tissue metabolism, mirroring early responses to hemorrhage and/or rupture. Additionally, we found that the BLVRB, which had been previously proposed by Matic et al.^18^ to define IPH, was also contained in our multiomics model (component 4).

Literature search for the MULTI 1 elements indicated an overall relationship (15 of the total 22) to vascular smooth muscle function and inflammation in cardiovascular diseases (Table S8). Interestingly, the regulatory network based on the dominant component, identified SRF as the driving factor in peri‐rupture remodeling. Despite the fact that a small media could not be excluded from the dissected tissue during the CEA surgery, the lack of correlation between the dominant component and the media size (Figure 4A) indicated the SRF‐driven network was only associated with intimal not medial processes related to IPH. SRF is a MADS family type TF, which acts by binding CArG box motifs. The relevance of SRF for cardiovascular health is well documented.^48^, ^49^ In cardiac and vascular smooth muscle cells, it was seen to drive phenotypic switch by regulating contractile gene programs.^50^ In endothelial cells, SRF is essential for VEGF‐induced cell signaling and angiogenesis, and thus endothelial dysfunction.^51^ Moreover, SRF mediates cellular lipid and glucose responses by controlling LXRB gene expression, modulating these metabolic sensors.^52^ While a direct role in ischemic heart disease is likely, experimental data to underpin this notion is lacking.

Mechanistically, SRF transcriptional responses depend critically on its coactivator. The major coactivator classes are T‐cell factors (e.g., TCF21,^53^ ELK1/2), which are responsible for renin–angiotensin–aldosterone system (RAAS)‐activated mechanosensing, and myocardin‐family members (e.g., MRTF), which transduces mothers against decapentaplegic homolog (SMAD) and Rho‐associated kinase responses.^54^ As pointed out by Gualdrini et al., competition between T‐cell and myocardin family members for SRF will skew its responses toward antagonistic proliferative and contractile programs of gene expression, respectively.^55^ Our data seem to plead for MKL1/2‐SRF signaling as the major dysregulated axis in IPH plaque, suggesting that remodeling responses will prevail.

Although our integrative multiomics approach holds promise, it has a number of limitations. First, this study was based on a moderately dimensioned cohort study containing CEA tissues from male patients and may therefore only partly reflect the population's diversity in disease risk profile as well as pathogenesis. The fact that we were able to confirm the validity of our selected genes in multiomics model in several other cohorts, however, indicates that our findings have a wider scope. Second, the multiomics approach was only based on genes, proteins and peptides. Incorporation of metabolomics and lipidomics into the multiomics signature may further strengthen the model and deepen our insight into key processes in this clinically relevant stage transition.^56^, ^57^

## 5 CONCLUSIONS

In conclusion, our study underpins the added value of integrative multiomics analysis to a single‐omics study of human atherosclerosis. Comparing a single‐ and multiomics analyses of human carotid atherosclerotic plaque, we showed that while both approaches perform excellently in segregating low‐ from high‐risk plaques, the latter provides much deeper insight into critical processes taking place prior to or just after plaque rupture in humans. Moreover, our integrative multiomics approach revealed an SRF‐driven gene/protein network, associated with this phase transition, which could guide efforts to the design of new treatments to promote non‐expansive plaque healing.

## CONFLICT OF INTEREST

The authors declare that there is no conflict of interest.

## ETHICS APPROVAL AND CONSENT TO PARTICIPATE

All patient materials were collected in the Maastricht Pathology Tissue Collection in line with the Dutch Code for Proper Secondary Use of Human Tissue (https://www.federa.org) and the local Medical Ethical Committee (protocol number 16‐4‐181). This study conforms to the Declaration of Helsinki, all participants have given informed written consent prior to the inclusion.

## AUTHOR CONTRIBUTIONS

Han Jin conceived the study, processed the data, performed the computational analyses, and drafted and revised the manuscript with input from all co‐authors. Cornelis J.J.M. Sikkink and Barend M.E. Mees performed the CEA surgery. Wouter Eijgelaar, Marco Manca, and Mat J.A.P. Daemen collected the plaque samples. Peter Juhasz performed proteomics and peptidomics experimental analysis. Immunostaining was performed by Judith C. Sluimer, Kim van Kuijk, Olivia Waring, Gregorio E. Fazzi, Marion J.J. Gijbels, and other experienced colleagues. Marco Manca helped in data analysis and multiomics data alignment. Joël M.H. Karel, Evgueni Smirnov, Martina Kutmon, and Chris T.A. Evelo gave input in the data analysis strategies. Ljubica Matic and Ulf Hedin performed analysis on the BiKE cohort dataset. Judith C. Sluimer, Pieter Goossens, Ljubica Matic, Marco Manca, and Olivia Waring offered critical revision. Erik A.L. Biessen conceived and supervised the study, provided funding and critical feedback at all stages.

## Supporting information

Supporting Information

Supporting Information

Supporting Information

Supporting Information

Supporting Information

Supporting Information

Supporting Information

Supporting Information

Supporting Information

Supporting Information

Supporting Information

Supporting Information

Supporting Information

Supporting Information

## Data Availability

All microarray data are available in public data repositories. MaasHPS transcriptomics can be accessed from the Gene Expression Omnibus (GEO, https://www.ncbi.nlm.nih.gov/geo/) with accession number GSE163154. BiKE transcriptomics can be accessed from the GEO with accession number GSE21545. The other two validation data can be accessed from the GEO with accession numbers GSE43292 and GSE28829.
