# Supplementary material for: Integrative multiomics analysis of human atherosclerosis reveals a serum response factor‐driven network associated with intraplaque hemorrhage
Source: Clin Transl Med. 2021 Jun 27;11(6):e458. doi: 10.1002/ctm2.458 (PMC8236116; doi:10.1002/ctm2.458)
Supplement: Supplementary file 1 — Supporting Information [file CTM2-11-e458-s005.pdf]

Figure S1

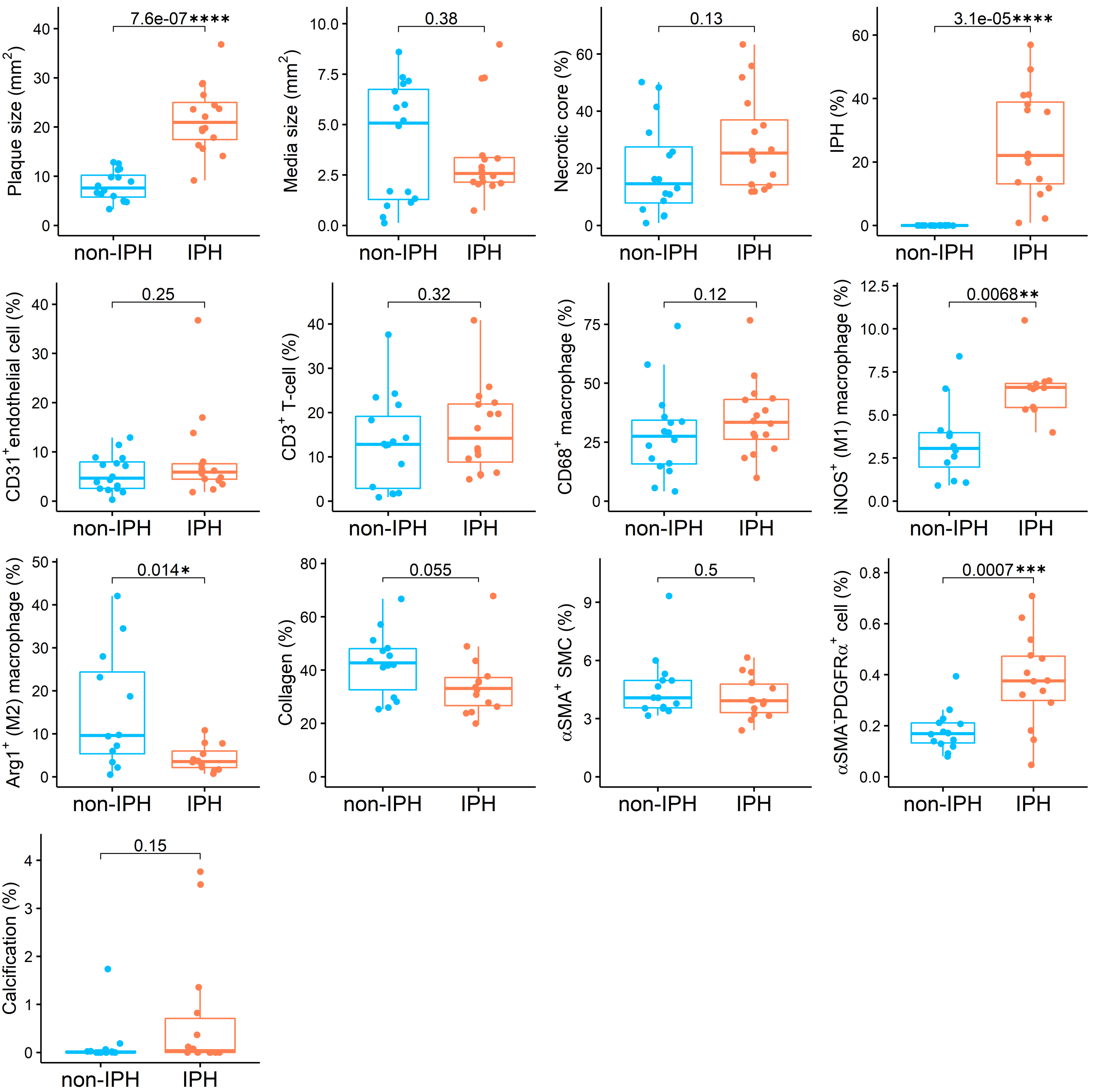

Figure S1 Distribution of plaque traits

Distribution of plaque traits between paired non-IPH and IPH (both n = 16) samples.
