# Supplementary material for: Integrative multiomics analysis of human atherosclerosis reveals a serum response factor‐driven network associated with intraplaque hemorrhage
Source: Clin Transl Med. 2021 Jun 27;11(6):e458. doi: 10.1002/ctm2.458 (PMC8236116; doi:10.1002/ctm2.458)
Supplement: Supplementary file 2 — Supporting Information [file CTM2-11-e458-s006.pdf]

Figure S2

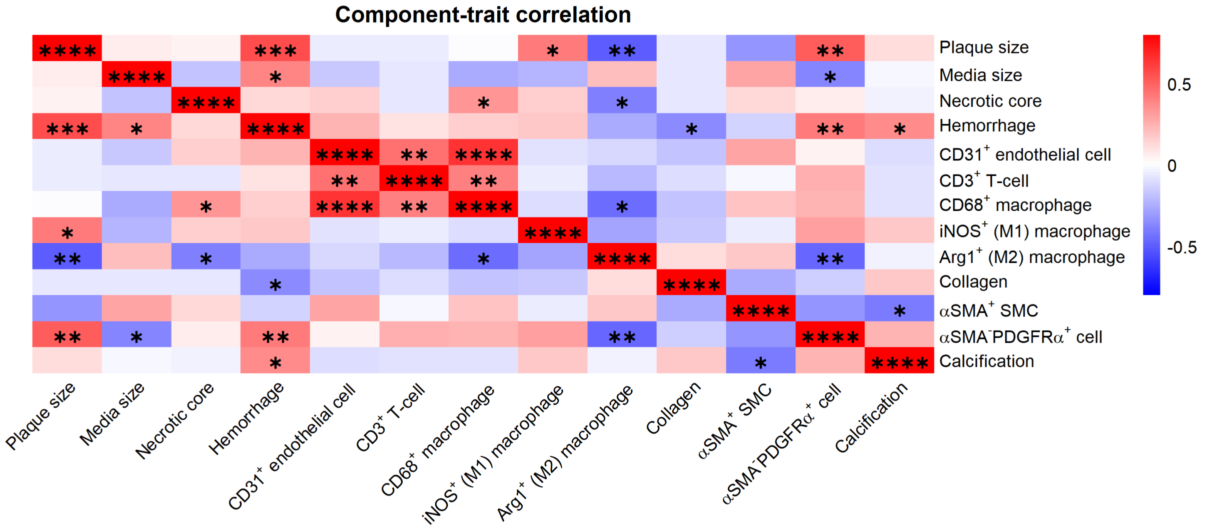

Figure S2 Correlations between plaque traits

Heatmap showing correlations between plaque traits.
