# Supplementary material for: Integrative multiomics analysis of human atherosclerosis reveals a serum response factor‐driven network associated with intraplaque hemorrhage
Source: Clin Transl Med. 2021 Jun 27;11(6):e458. doi: 10.1002/ctm2.458 (PMC8236116; doi:10.1002/ctm2.458)
Supplement: Supplementary file 3 — Supporting Information [file CTM2-11-e458-s011.pdf]

Figure S3

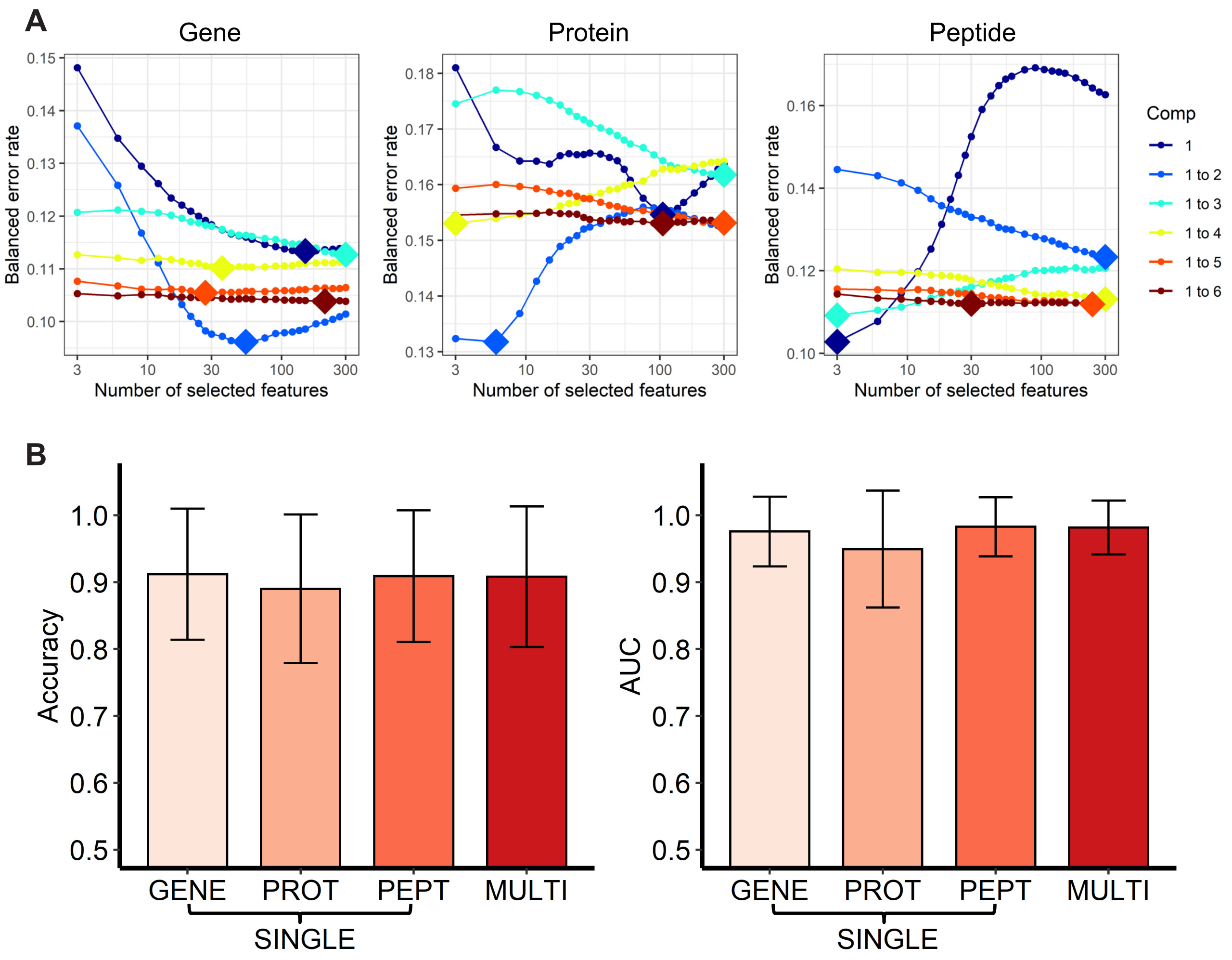

Figure S3 Parameter optimization

(A) Balanced Error rate of training models under different parameter settings for single-omics sPLS-DA on transcriptomics, proteomics, and peptidomics. A range of features from 3 to 300 (from 3 to 30 in steps of 3, from 30 to 60 in steps of 6, from 60 to 150 in the step of 15, and from 150 to 300 in the step of 30) was set for each component. Results were calculated with stratified 5-fold cross-validation and 1,000 random repeats. (B) Performance of the prediction models under the optimal parameter settings. Results were measured by accuracy and AUC, with stratified 5-fold cross-validation and 10,000 random repeats. Results are presented as mean ± SD. GENE, PROT, PEPT stand for the results of single sPLS-DA (SINGLE) based on transcriptomics, proteomics, and peptidomics, respectively. MULTI represents the results of integrative sPLS-DA on the three omics.
