# Supplementary material for: Integrative multiomics analysis of human atherosclerosis reveals a serum response factor‐driven network associated with intraplaque hemorrhage
Source: Clin Transl Med. 2021 Jun 27;11(6):e458. doi: 10.1002/ctm2.458 (PMC8236116; doi:10.1002/ctm2.458)
Supplement: Supplementary file 4 — Supporting Information [file CTM2-11-e458-s009.pdf]

Figure S4

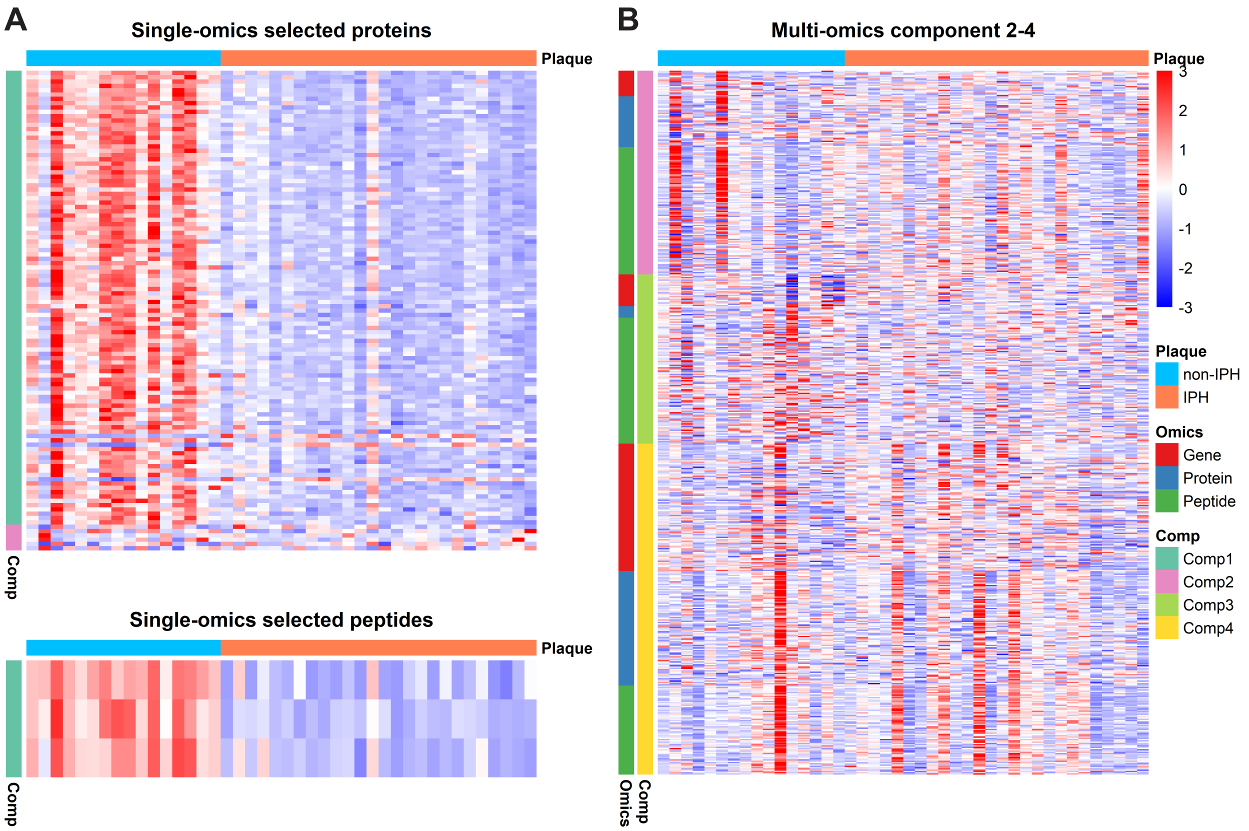

Figure S4 Heatmap of the selected features

(A) Heatmaps showing the expression level of the selected proteins from proteomics and peptides from peptidomics by single-omics analysis. (B) Heatmap showing the expression level of the selected features for component 2–4 of multi-omics analysis. Expression values were z-normalized per row.
