# Supplementary material for: Integrative multiomics analysis of human atherosclerosis reveals a serum response factor‐driven network associated with intraplaque hemorrhage
Source: Clin Transl Med. 2021 Jun 27;11(6):e458. doi: 10.1002/ctm2.458 (PMC8236116; doi:10.1002/ctm2.458)
Supplement: Supplementary file 5 — Supporting Information [file CTM2-11-e458-s001.pdf]

Figure S5

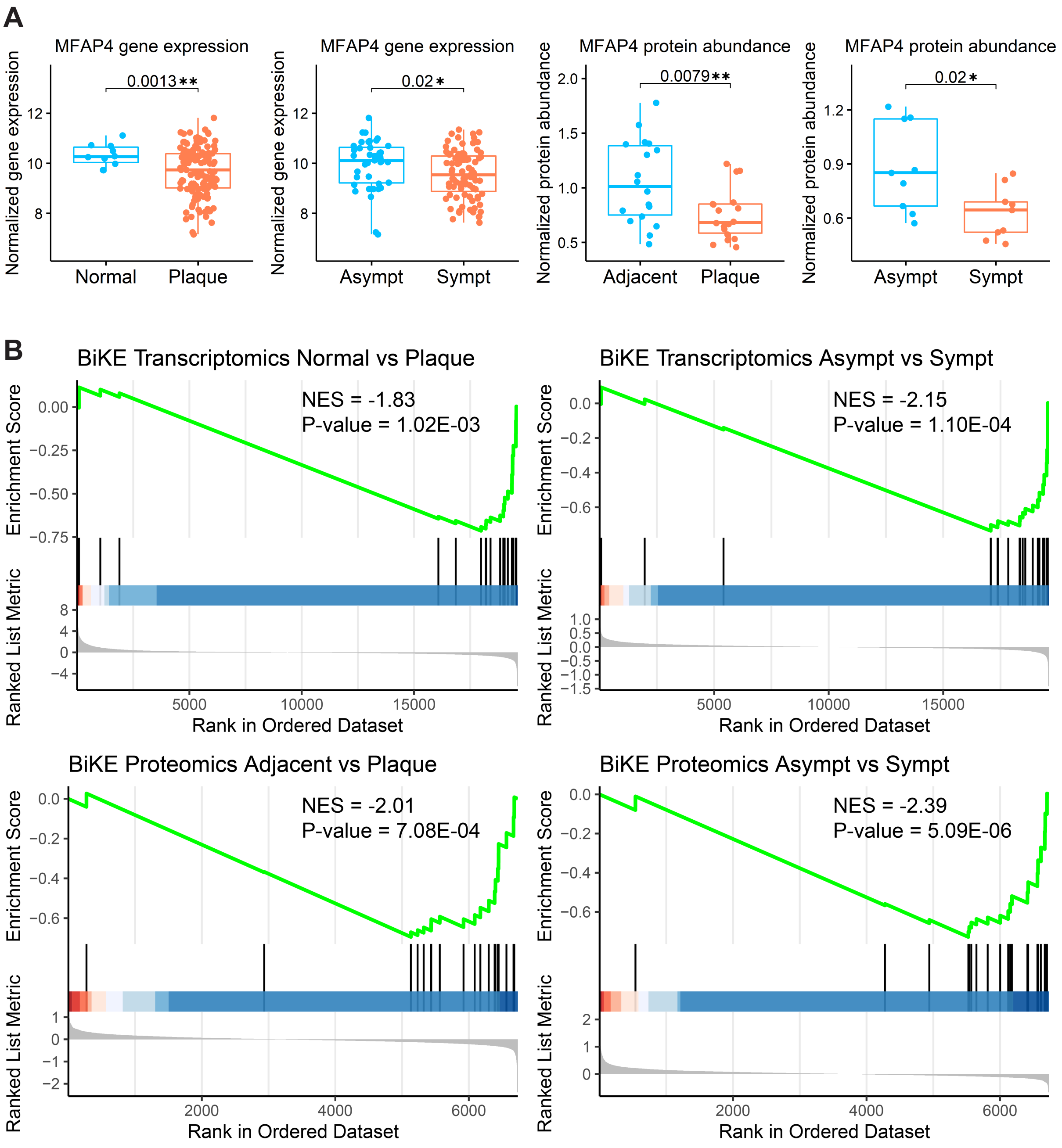

Figure S5 Validation of MFAP4 and other SRF targeting genes in BiKE cohort

(A) Gene expression and protein abundance of MFAP4 in BiKE cohort. The levels of gene expression were compared between normal (Normal, n = 10) and atherosclerotic arteries (Plaque, n = 127), and between asymptomatic (Asympt, n = 40) and symptomatic (Sympt, n = 87) patients. The levels of protein abundance were compared between adjacent healthy segments (Adjacent, n = 18) and atherosclerotic segments (Plaque, n = 18), and between asymptomatic (Asympt, n = 9) and symptomatic (Sympt, n = 9) patients. Results are shown as box and whiskers plots with the same setting in Figure S1. P-values were calculated using a two-tailed Wilcoxon rank-sum test or Student's t-test. (B) GSEA of the SRF targeting network members (see Figure 4B) based on BiKE cohort. Genes and proteins in BiKE transcriptomics and proteomics were ranked based on the comparisons in Figure S5A and were used as the GSEA background. NES, normalized enrichment score.
