# Supplementary material for: Integrative multiomics analysis of human atherosclerosis reveals a serum response factor‐driven network associated with intraplaque hemorrhage
Source: Clin Transl Med. 2021 Jun 27;11(6):e458. doi: 10.1002/ctm2.458 (PMC8236116; doi:10.1002/ctm2.458)
Supplement: Supplementary file 6 — Supporting Information [file CTM2-11-e458-s003.pdf]

Figure S6

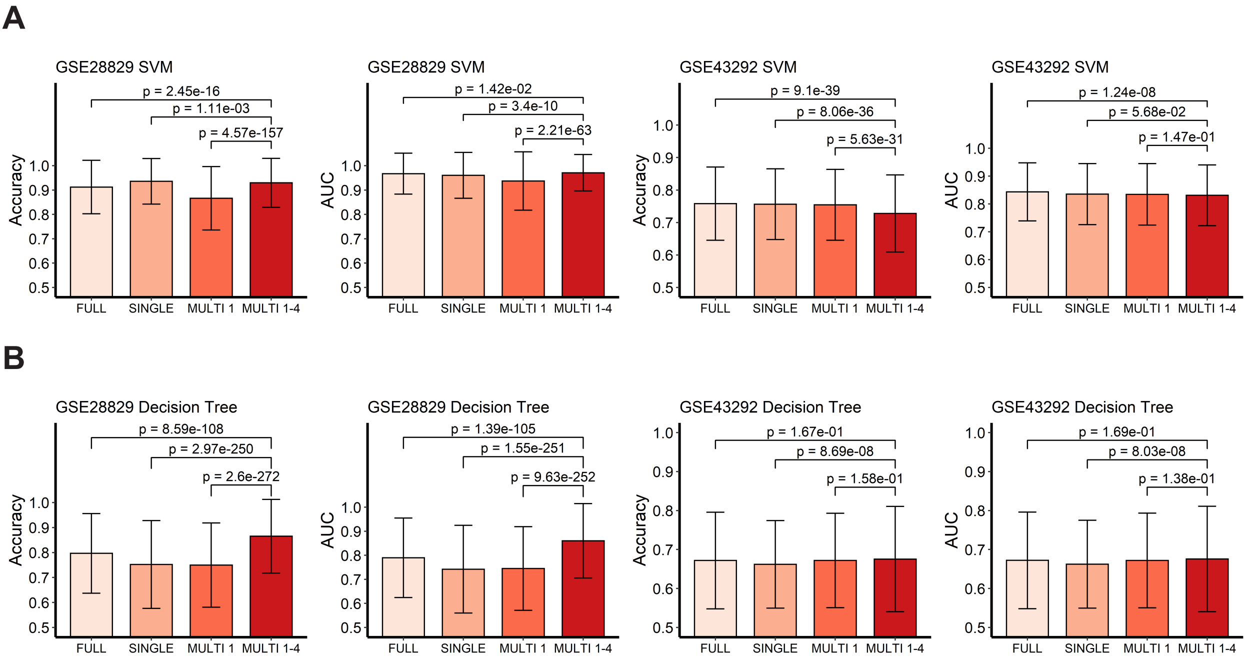

Figure S6 Validation of selected gene sets in independent cohorts

Classification performance based on single-omics (SINGLE), multi-omics component 1 (MULTI 1), and multi-omics component 1-4 (MULTI 1-4) feature subsets as well as the full data (FULL) as a baseline on two independent cohorts GSE28829 and GSE43292. Results were measured by accuracy and AUC using SVM (A) and decision tree (B), respectively, with stratified 5-folds cross-validation and 1,000 random repeats. Results are presented as mean ± SD.
